# Impact of land transformation, management and governance on subjective wellbeing across social–ecological systems

**DOI:** 10.1007/s11625-024-01584-5

**Published:** 2024-12-12

**Authors:** Patricia Santillán-Carvantes, Alejandra Tauro, Patricia Balvanera, Juan Miguel Requena-Mullor, Antonio J. Castro, Cristina Quintas-Soriano, Berta Martín-López

**Affiliations:** 1 Faculty of Sustainability, Social-Ecological Systems Institute (SESI), Leuphana University, Lüneburg, Germany; 2 Instituto de Investigaciones en Ecosistemas y Sustentabilidad, Universidad Nacional Autónoma de México, Morelia, México; 3 El Colegio de Puebla AC, Puebla, Mexico; 4 Cape Horn International Center for Global Change Studies and Biocultural Conservation (CHIC), Universidad de Magallanes, Puerto Williams, Chile; 5 Department of Biology and Geology, Andalusian Center for Global Change-Hermelindo Castro (ENGLOBA), University of Almeria, La Cañada de San Urbano, 04120 Almeria, Spain

**Keywords:** Subjective wellbeing, Global south, Social-ecological systems, Content analysis

## Abstract

Recognizing and understanding the subjective wellbeing (SWB) of individuals is essential for designing effective policies that promote human development and the sustainable management of social-ecological systems (SES). This is particularly important for smallholders, critical stewards of biodiversity who face acute livelihood challenges. This article explores how smallholders inhabiting tropical dry forests in Mexico perceive their SWB and how it changes across a spectrum of SES that undergo different land transformations, management intensities, and governance dynamics. Our aims are to identify the dimensions of SWB that smallholders perceive, understand how these dimensions change across SES, and examine how smallholders’ perceptions of fulfilled material and non-material dimensions vary across SES. We analyzed the content of 25 in-depth interviews with farmers and identified 48 SWB items belonging to six categories: (1) social capital, (2) economic capital, (3) agency, (4) nature, (5) pleasant non-work activities, and (6) governmental services, and two additional dimensions referred to obstacles and enablers. We found two prevailing visions of SWB: ‘living well’ prevails especially in areas with communal governance and medium management intensity, and ‘need to earn more’ prevails in areas with individual governance and intensified land management. As management is intensified and governance fosters individualism, the lower the self-perceived material and non-material satisfaction. We discuss the different SWB found per SES, as well as strategies that can foster smallholder’s SWB and SES dynamics that can motivate different conservation goals and sustainable uses of nature, especially in biodiverse areas.

**Supplementary Information:**

The online version contains supplementary material available at 10.1007/s11625-024-01584-5.

## Introduction

Recognizing and understanding the subjective wellbeing (SWB) of individuals is essential for designing effective policies that promote human development and the sustainable management of social-ecological systems (SES). Yet, there is still little knowledge on the assessment of SWB of smallholders associated with human-nature relationships (Delgado et al. 2019; Nowak-Olejnik et al. 2022), particularly in the Global South. Smallholders in the Global South are the stewards of a large fraction of the world’s biodiversity (Díaz et al. 2015). Despite their important role for preserving biodiversity, they also face acute challenges, including unequal access to markets and incentives for agriculture production, welfare gaps, limited labor opportunities and availability, and increased violence and crime (Bartra 2002). Yet, in places such as Latin America, reported happiness is higher relative to their income levels than in other nations worldwide (Rowan 2023). Thus, a better understanding of the variables that contribute the most to determining smallholders’ SWB, results in critical decision-making processes (Tauro et al. 2018; IPBES 2022; Begho and Odeniyi 2024).

One definition of SWB is “a person’s cognitive and affective evaluations of his or her life; these evaluations include emotional reactions to events as well as cognitive judgments of satisfaction and fulfillment” (Diener et al. 2002, p. 63). National programs began to standardize and quantify SWB with national ‘barometers’ such as the Australian Unity Well-being Index (McGregor et al. 2009), or the developing notion of *Buen Vivir* (‘living well’) in Latin America that use quantitative methods to measure SWB rooted in the local culture (Guardiola and García-Quero 2014; Sterling et al. 2017). Moreover, national programs, such as Bhutan’s Gross National Happiness Index, go beyond wellbeing measures mainly based on income and attempt to measure a holistic wellbeing (Sterling et al. 2017). Despite these measurements, and the growing recognition of SWB in research and policy, there are still two main gaps in relation to the understanding of SWB (Diener et al. 2018). First, much research and programs have been conducted in North America and Europe, leaving the Global South underrepresented. Second, the majority of SWB research has relied on self-report surveys that still fail to identify the SWB diverse visions and their levels of satisfaction in local contexts. In fact, the Intergovernmental Platform on Biodiversity and Ecosystem Services (IPBES) has made a call to develop research that sheds light on how disparate visions of wellbeing can motivate different conservation goals and sustainable uses of nature (Díaz et al. 2015).

Overall, there is consensus on the inextricable link between nature and people’s wellbeing (MA 2005; Díaz et al. 2015). These have been assessed through the benefits people obtained from nature, using concepts such as ecosystem services (MA 2005) and Nature’s Contributions to People (NCP) (Díaz et al. 2018). Some have hypothesized that more interaction with nature will lead to happier and healthier people (Annerstedt and Währborg 2011; Russell et al. 2013), and yet, there are global observations that show an increase in human wellbeing with declining trends of biodiversity and NCP (MA 2005; Raudsepp-Hearne et al. 2010). The so-called “environmentalist’s paradox” hypothesized that incomplete wellbeing measures could explain this contradiction, as well as the excessive focus on material dimensions of wellbeing or the fulfillment of wellbeing through technological innovations (Raudsepp-Hearne et al. 2010). Indeed human-nature relationships and, thus the wellbeing derived from them, are shaped by various ecological, social, cultural, and governance drivers across scales (Berkes et al. 1998; Chapin et al. 2009; Ostrom 2009; Martín-López et al. 2017). Yet, it is not clear the impact these drivers have on the way people make decisions and manage nature to fulfill their material and non-material desires across social-ecological systems (Norström et al. 2022). In fact, to date, large knowledge gaps remain at the interface between wellbeing, in particular SWB, and the different social-ecological contexts (Mastrángelo et al. 2019), especially in Latin America.

Latin America, with many hotspots for biodiversity (Toledo 1999), faces major sustainability challenges, as the transformation of land cover for productive activities affects not only biodiversity but also the smallholders’ wellbeing (Nagendra 2018). Tropical dry forests (TDF) are considered biodiversity hotspots and, at the same time, are one of the most threatened ecosystems in the world (Janzen 1988; Sánchez-Azofeifa et al. 2005; Portillo-Quintero et al. 2015). Smallholders are the stewards and managers of a large fraction of biodiversity and productive lands in Latin America and especially in TDF (Boege 2009; Graeub et al. 2016; Bellon et al. 2021; Brondizio et al. 2023), but the impact of land management on their SWB has not been explicitly addressed. Considering the perceptions of smallholders in TDF according to different social and ecological conditions is most urgently needed (Quijas et al. 2019), particularly the effect of land cover transformation, land management intensity, and governance that shape the dynamics of SES (Santillán-Carvantes et al. 2023) with an impact on smallholders’ SWB.

This research explores the questions of how subjective wellbeing of smallholder land managers is perceived in a TDF in Mexico, and how these perceptions differ across SES representing diverse land transformations, management intensity, and governance, and what are the dimensions of SWB perceived and how the material and non-material dimensions are fulfilled? Specifically, we aim to: (1) identify the dimensions of SWB that smallholders perceive, (2) understand how these dimensions change across different SES types, and (3) examine how smallholders’ perceptions of fulfilled material and non-material dimensions varied across SES types.

## Methods

### Case study

**Fig. 1:**
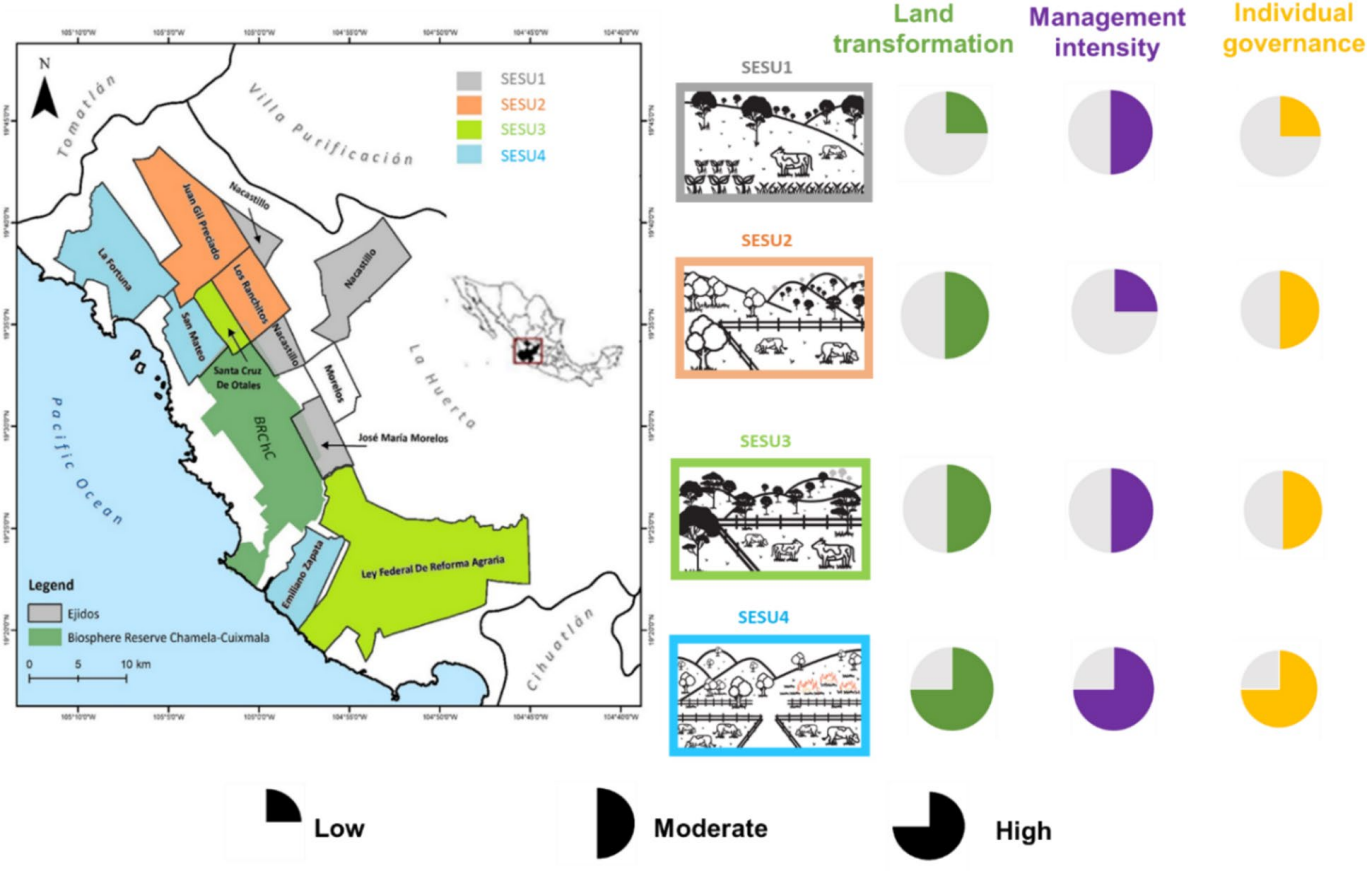
Location of the Chamela-Cuixmala region, comprised of the Biosphere Reserve Chamela-Cuixmala (BRChC; dark green color) and the transition areas in the governance units (*ejidos*) in the adjacent area in the Municipality of La Huerta and Villa Purificación, Jalisco, Mexico. Different colors of the *ejidos* denote different social-ecological system units (SESU, Santillán-Carvantes et al. 2023). The area covered by the colored circles denotes the intensity of land transformation, management intensity, and individual governance in each SESU

The Chamela-Cuixmala region is part of the TDF biome along the Mexican Pacific coast (Ceballos and García 2010). The region comprises the Chamela-Cuixmala Biosphere Reserve and its transition area (UNESCO 2022), located in the municipalities of La Huerta and Villa Purificación in Jalisco, Mexico (Fig. 1). The ownership regime is a critical factor that affects the landscape configuration of transition areas. Most of the land (70–80%) is under a specific governance unit in Mexico, called *ejido*, a semi-communal land tenure regime that emerged from the land redistribution policies following the Mexican Revolution of the 1910s (Castillo et al. 2005; Pingarroni et al. 2022). Local collective management arrangements have been developed in many *ejidos* and are operationalized through an *ejidal* assembly (Toledo 1996; Agrawal 2007; Schroeder and Castillo 2013). Land tenure within *ejidos* in this region can occur in three ways (Schroeder and Castillo 2013). First, *ejidatarios*, or the landholding members of the *ejido*, can inherit the land right (*ejidal* plots), sell it, and vote in the *ejidal* assembly to take communal decisions. *Ejidatario* has rights over communal lands within the *ejido*. Second, *posesionarios* possess land within the *ejido* but cannot pass it on to the following generation. *Posesionarios* do not have rights on communal lands and cannot vote in the *ejidal* assembly. Each *ejido* determines the level of *posesionarios* participation in collective management. Finally, *avecindados* are those who have settled within the *ejido* for more than a year and possess neither land rights nor vote in the Assembly (Schroeder and Castillo 2013). Traditionally, men hold most of these three types of land rights and make land-related decisions, although there are few “*ejidatarias*” (women). In this work, we focused only on *ejidatarios* (i.e., men smallholders) in the surrounding areas of the Chamela-Cuixmala biosphere reserve.

*Ejidatarios* within the *ejidos* surrounding the Chamela-Cuixmala biosphere reserve have been undertaking extensive cattle ranching as well as silvopastoral activities (Tauro et al. 2021; Sánchez-Romero et al. 2021). Cattle ranching is strongly limited by biophysical aspects, such as water availability, and economic ones, such as financial resources to invest in cattle maintenance (Maass et al. 2005). Distinct social-ecological system units (SESU) have been identified in the area based on biophysical conditions (i.e., topography, soil fertility, carbon stock, etc.), land transformation (i.e., land cover), land management intensity (i.e., cattle rotation, number of cattle, number of wood extraction, etc.) and governance characteristics (i.e., individual land tenure by *avecindados*, or communal land tenure by *ejidatarios*) (Santillán-Carvantes et al. 2023) (Fig. 1). The four SESU showed a geographical gradient from the country’s center (SESU1) to the coast (SESU4) aligned with topographic and climatic conditions. Furthermore, the different SESU represented a gradient of land cover transformation (from SESU1, the least transformed, to SESU4, the most transformed), management intensity (from SESU2, which is the most extensively managed to SESU4, the most intensively managed) and governance systems (from SESU1 that mostly implemented communal governance to SESU4, in which the governance system primarily relies on private land and individual land tenure) (Fig. 1).

### Data collection

To study the perceptions of smallholders about SWB in the Chamela-Cuixmala region, we used 25 semi-structured in-depth face-to-face interviews conducted between February and June 2015 (Tauro et al. 2018). The participants were chosen to represent the composition of the *ejidal* assembly. *Ejidatarios* (men smallholders) in the community have historically been given property rights; they are in charge of the land and engage in productive management activities (Lazos-Chavero et al. 2016). The rural family model continues to encourage traditions where male ownership and management of property predominate, despite the fact that since 1992, the law has allowed women to acquire land (Almeida 2012; Vázquez-García 2015; Tauro et al. 2018). For this study, we focused only on those who undertake cattle ranching. We thus focused only on the most common type of *ejidatario*: these were only men, the youngest being 35 years old, and half of them being at least 60 years old (see Table S2). Two to seven *ejidatarios per ejido* were interviewed. Because of the scarce representation of young *ejidatarios* and women in the *ejidal* assembly and among cattle ranchers, we chose to emphasize the differences among the SESU for the most common type of ejidatario, rather than on the heterogeneity within each ejido. This approach ensures that our sampling strategy accurately reflected the current demographic and occupational structure of the *ejidatarios* in the area.

The interview’s guiding questions to unravel the SWB of the respondents were related to their current and desired SWB. Concerning the current SWB, we asked How do you feel today with your life and why? Do you feel satisfied with your life? What do you do to have a “good life”? Concerning questions on desirable SWB we asked what is a good life? What are your wishes for a good life in 10 or more years? For you to be better, what would you change and why? And what would you keep the same and why?

The responses were analyzed through a deductive-inductive content analysis. First, researchers used a deductive approach following the wellbeing dimensions proposed by Rogers et al. (2012). Second, researchers added new categories for the most typical items that did not fit any of the proposed dimensions. This process was repeated until the final coding scheme was agreed upon. Only one person coded all the responses to reduce intercoder bias. Rogers et al. dimensions include *material living standards, Health, Physical and economic security, Ecosystems, Education, Work and leisure, Agency and political voice,* and *Social relationships*. *Material living standards* were related to housing, food, and public and private services (i.e., having a car). *Health* was related to medical visits or self-perceptions of their health. *Physical and economic security* was related to economic capital and the sense of physical security (i.e., non-violent contexts). The dimension of *ecosystems* was related to the biotic or abiotic factors within an ecosystem (i.e., water, cattle, or geographic features) that contribute to their SWB. *Education* was related to access to school or learning activities. We coded for education, whenever the person mentioned something related to his education or his family as illustrated by the quotes “The most beautiful thing about a family is giving study to the family”, “I work, because I couldn’t study, then what am I going to work on?”. The dimension of *work* was related to having a meaningful occupation and the security of having an income, while the dimension of *leisure* referred to the satisfaction gained with activities outside of work. *Agency and political voice* were related to perceptions of having a voice in decision-making, trust in government, free mobility, and freedom of choice. *Social relationships* were related to social interactions, community, sense of place, traditions, generations, and religious activities. A response could be coded for several SWB dimensions. For example, the response to how you feel today with your life: “*I feel well and complete with my family, I work independently, without schedule. I feel healthy and strong*” (S4) was interpreted as social relationships, a sense of plenitude, good health, and agency with the sense of freedom. As mentioned before, after the first classification of the responses, we found that some answers did not match with the given dimensions from Rogers et al. (2012), for instance, sadness or the sense of decadence. Therefore, we developed a locally relevant classification of SWB dimensions. In this process, we merged items and added new inductively revealed dimensions, for instance, the enablers and obstacles for a good SWB. The authors inductively coded these two last ones due to the inherent difference in conception between the perceived items of SWB and obstacles and enablers mentioned by the interviewees. We finally used six dimensions of SWB -i.e., social capital, economic capital, agency, nature, pleasant non-work activities, and government and services. The item “pleasant non-work activities” was named this way to indicate that it includes more activities than those associated with traditional leisure activities such as outdoor activities, going to the cinema or playing sports and included, for instance, spending time with the family, having fun, or going to their paddock as a distraction. We analyzed the frequency of mentions for each of these dimensions and for each of the items within these dimensions.

### Change in perceptions across the Social-Ecological System Units (SESU)

Each of the 25 interviewees was associated with one of the four SESU (2–7 *ejidatarios* interviewed per SESU). With the frequency of mentions per dimension per SESU, we checked which data followed normal distributions through the Shapiro–Wilk test (Shapiro and Wilk 1965), and we tested for differences among SESU by using ANOVA. When frequencies of the SWB dimensions did not follow a normal distribution, we ran Kruskal–Wallis tests. The Kruskal–Wallis test is preferred for small sample sizes because it does not rely on the assumption of normality and is robust to deviations from normality. This makes it suitable for situations where accurately estimating the data distribution is challenging, ensuring the validity of the results even with limited data (Siegel and Castellan 1988). Post hoc tests were implemented when significant differences between SESU were identified, using a Tukey test (since only normal data presented significant differences). To test for differences between current and desirable SWB, we used a t-test and a Mann–Whitney test. All analyses were carried out using R version 4.0.5 (R core Team 2014). We used the packages “car” (Fox and Weisberg 2019) for the ANOVAs, and the package “multcomp” (Hothorn et al. 2008) for the post hoc tests. The t-test and the Mann–Whitney test were part of the general commands of R.

### Fulfillment of material and non-material dimensions

In the content analysis, we distinguished whether or not the smallholders referred to material or non-material dimensions and whether those dimensions were perceived as fulfilled or not. Material dimensions referred to tangible needs, such as food or financial capital. Non-material dimensions referred to intangible needs like health or social relationships. Fulfilled referred to when the interviewee expressed satisfaction with the dimension, for example ‘*We have good food*’ (S1). In contrast, non-fulfilled referred to when the interviewee expressed dissatisfaction, for example, “*I feel wasted and tired*” (S3). Sometimes, the same answer could mean both ‘fulfilled’ and ‘unfulfilled’ for different dimensions of SWB. For example, the response “*life is a function of external forces, I feel disappointed, insecurity generates my concern. Something is not working well, but you do not have to make your life miserable for that. Besides, I feel happy; I have my family*” (S20). In this case, the SWB dimension of public security was not fulfilled, while the SWB dimension that refers to having a family (i.e., a small community) was perceived as fulfilled. In this case, both aspects referred to non-material dimensions, and therefore, this statement was coded as both fulfilled and unfulfilled non-material dimensions. Moreover, one statement could refer to material and non-material dimensions as illustrated by the following answer: “*Very good, I feel complete, my family is complete, I work independently, there is no schedule, I am healthy*” (S4). In these cases, we coded the answer as material and non-material, and in this particular case as fulfilled material and non-material dimensions. Four variables were created from this content analysis: fulfilled and unfilled material dimensions and fulfilled and unfulfilled non-material dimensions. We then calculated their mean and standard deviation for each SESU and tested for significant differences between SESU using ANOVA and Kruskal–Wallis tests.

## Results

### Dimensions of subjective wellbeing

**Fig. 2:**
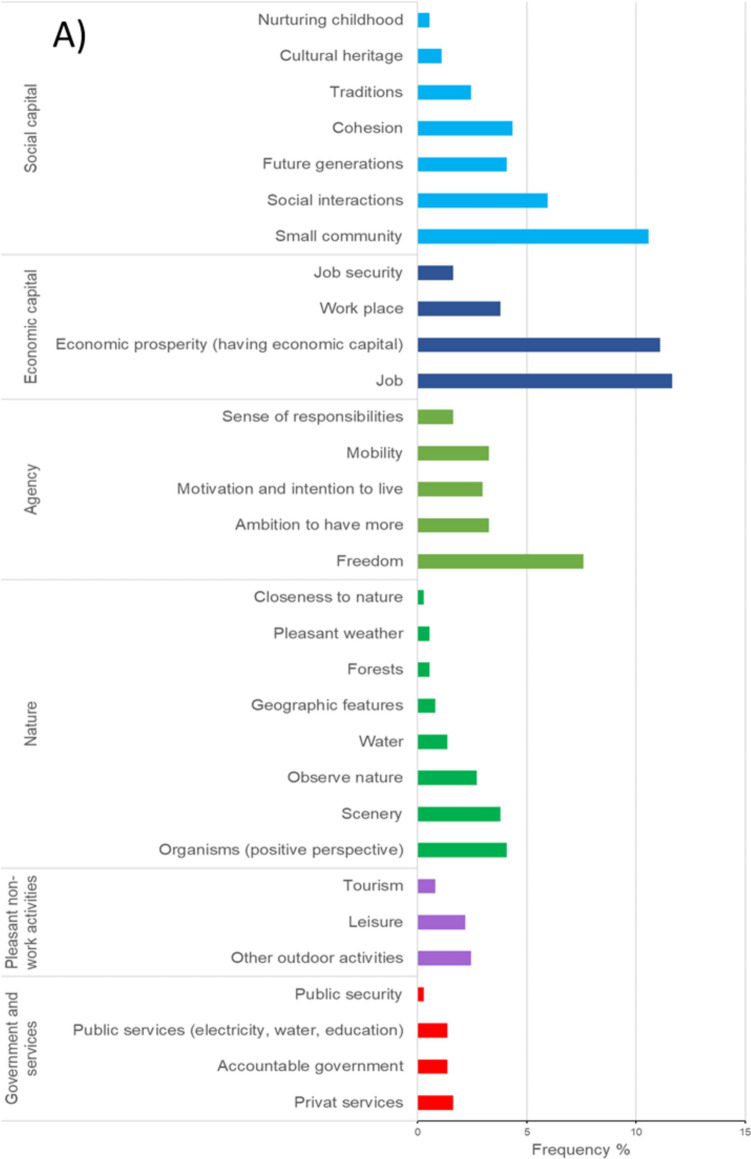

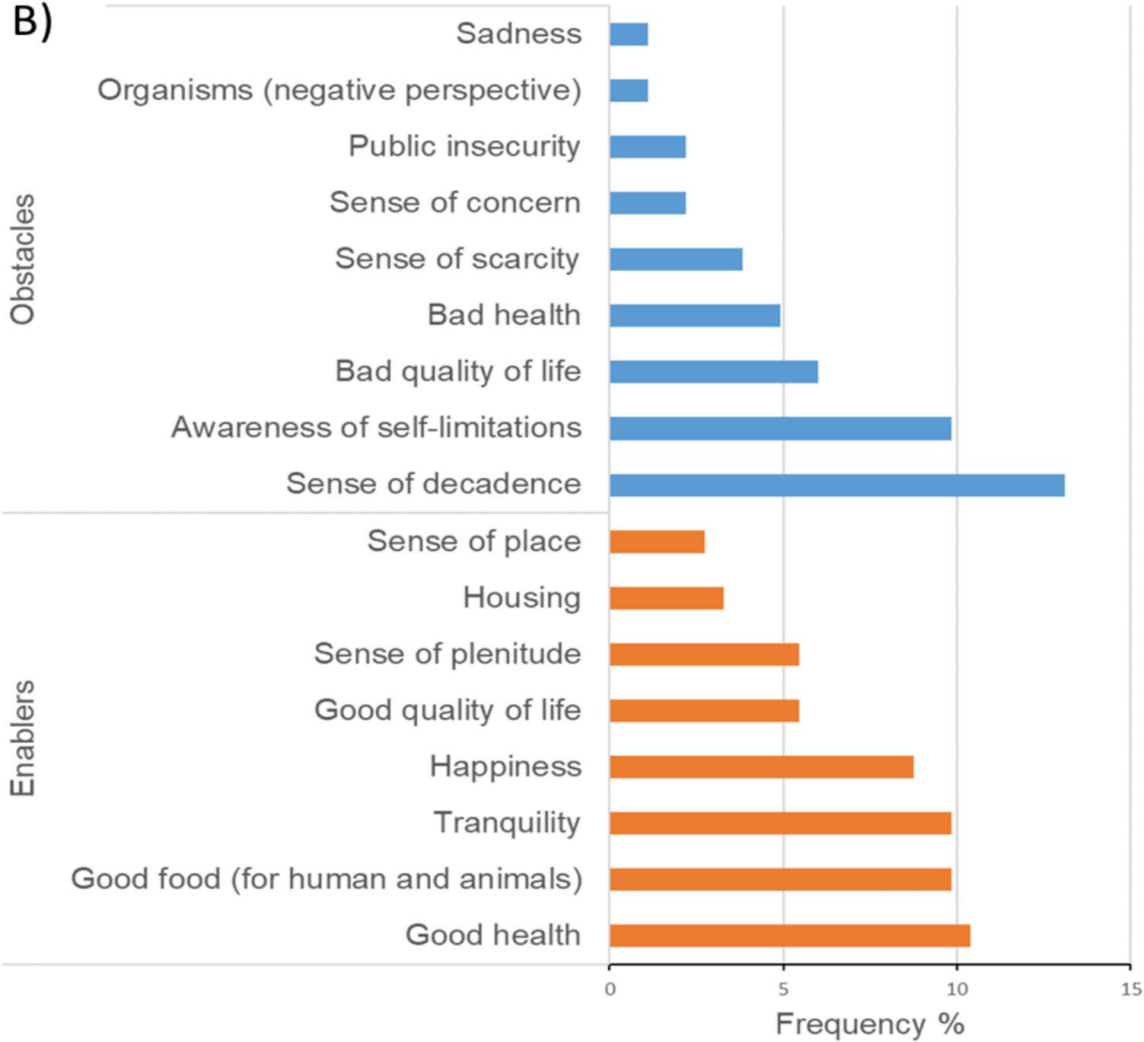
Relative frequency of **A** 31 items perceived from SWB and **B** 17 items of obstacles and enablers to a good SWB perceived by the 25 smallholders interviewed in the Chamela-Cuixmala region. The proportion of **A** was calculated from the total of 31 items mentioned (369), and the proportion of **B** was calculated from the total of 17 items mentioned (183)

The smallholders interviewed distinguished 48 different items related to SWB, which were organized into six dimensions of SWB, i.e., social capital, economic capital, agency, nature, pleasant non-work activities, and government and services (Fig. 2A) and into obstacles and enablers for achieving a good SWB (Fig. 2B).

Social capital and economic capital were the two most prominent dimensions of SWB (29% and 28% of all mentions, respectively, Fig. 2A). For social capital, social interactions and belonging to a small community were the most highly mentioned (6% and 11%, respectively, Fig. 2A). For example, people often pointed out the importance of the family in their lives, as illustrated by the following quote: “*I feel calm because my daughter supports me on my medical visits*” (S5, the complete details of the smallholders interviewed are found in table S3). Regarding economic capital, most respondents referred to economic prosperity and job aspects, as illustrated by the quote: “*Good, I have work and a source of income*” (S12). When referring to the job, some respondents expressed the satisfaction of having a meaningful occupation, as the following quote illustrates “*I feel satisfied; I achieved everything I wanted in my work*” (S25). The fourth most expressed item referred to the sense of freedom (7.5% of the statements), which represents the dimension of agency, often related to the freedom of their job, as illustrated by the following quote “*I work independently with no schedule*” (S4). Table S1 provides more examples of *verbatims* that represent the different items of SWB in each dimension.

We found two most prominent obstacles to achieving a good SWB: a sense of decadence and self-limitations. The sense of decadence (13% of all mentions; Fig. 2B) referred to the sense that there is no more space for wishes, as illustrated by the quote, “*There is no more good life to live in plenitude for people 50 or 60 years old, there is no space for wishes*” (S19) and “*I feel wasted, I cannot work because I get tired soon*” (S9). The self-limitations included the multiple obstacles faced (9.8%; Fig. 2B), for example, as illustrated in the quotes “*We have to be aware of what God brings to us*” (S18) and “*I did not know how to take advantage of the opportunities I had*” (S3).

The most prominent enablers of a good SWB included good health, good food, tranquility, and happiness (10%, 9.8%, 9.8%, and 8.7%, respectively; Fig. 2B). The quotes “*Thank God, the world owes me nothing, I asked God that my family do not get sick, and he gave it to me*” (S22), “*Good, I have work, and we eat nicely*” (S2), and “*I feel satisfied and happy, I achieve everything I wanted in my job*” (S25) are examples representing those enablers. Table S1 presents a selection of quotes for obstacles and enablers.

### Change in current and desirable subjective wellbeing across the Social-Ecological System Units (SESU)

**Fig. 3:**
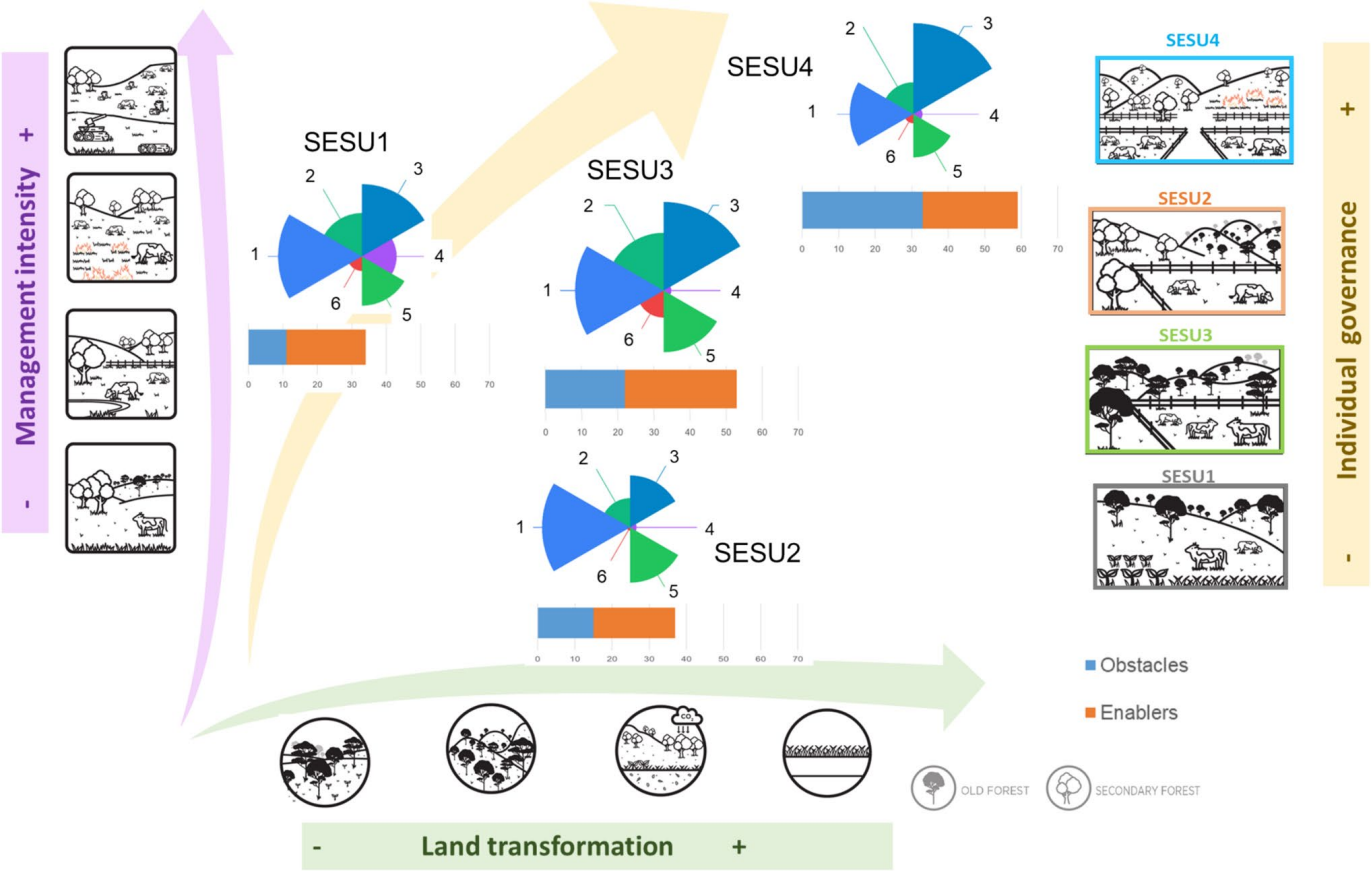
Current dimensions of subjective wellbeing (SWB) perceived across Social Ecological System Units (SESU): (1) Social capital. (2) Nature. (3) Economic capital. (4) Pleasant non-work activities. (5) Agency. (6) Government and services. The SESU are shown in an increasing gradient of land transformation (SESU1, SESU2, SESU3, and SESU4), management intensity (SESU2, SESU3, SESU1, and SESU4), and individual governance (SESU1, SESU3, SESU2, and SESU4). The length of the pie slice reflects the frequency of mentions of current SWB dimensions

The dimensions of SWB and the obstacles and enablers for a good SWB differed among SESU for both current (Fig. 3) and desirable (Table 1) conditions. As the land transformation increased (from SESU1 to SESU4), obstacles and enablers were mentioned more frequently in both current (obstacles: *X*^2^ = 4.53, df = 3, p-value = 0.209; enablers: *X*^2^ = 1.47, df = 3, p-value = 0.687, Table S3) and desirable conditions for a good SWB (obstacles: *X*^2^ = 2.86, df = 3, p-value = 0.413; enablers: *F-statistic* = 2.365, df = 3, p-value = 0.1, Table S4). As management intensity increased (from SESU2 to SESU4), nature was mentioned more frequently, and social capital was less frequently mentioned as desirable (Table 1; Table S5). As governance promoted communality (from SESU1 decreased to SESU4) and land transformation decreased (from SESU 4 to SESU1), pleasant non-work activities were more frequently mentioned as desirable (SESU1, Table 1).

**Table 1:**
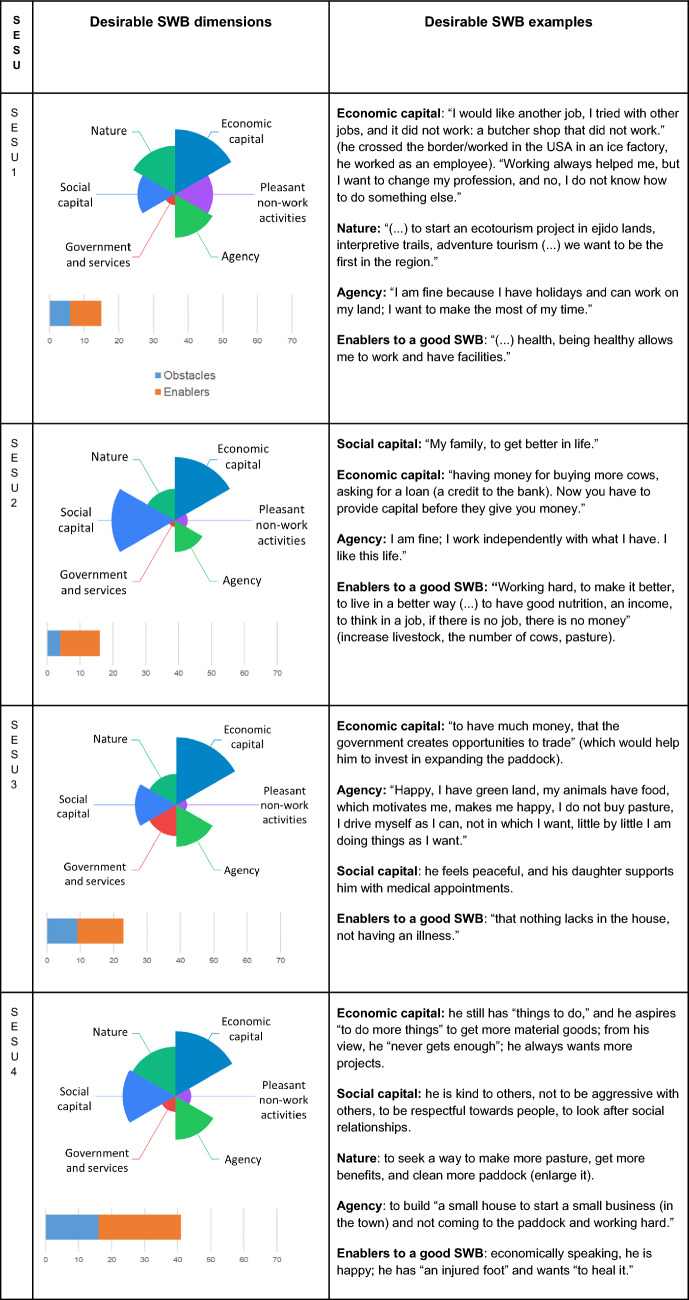
Changes in the desirable SWB dimensions across SESU

The length of the pie slice reflects the frequency of mentions of desirable SWB dimensions. Examples of the desirable SWB from the most frequent dimensions per SESU are presented to illustrate the concepts

With these results we observe a prevailing vision of “living well” in SESU with low to medium management and less land transformation (SESU1, SEU2, SESU3, Fig. 3) where we observed higher number of mentions of the dimensions “Nature”, and “Pleasant-non-work activities” and quotes such as “*how I live that is a good life, I go up and down as I want*” (S16, SESU1), or in the quote “*I am happy, I have a green area, my animals with food and that motivates me. It makes me happy. I don’t buy grass. I manage myself in what I can, not so much in what I want. Little by little I am doing things the way I want*” (S25, SESU3).

**Fig. 4:**
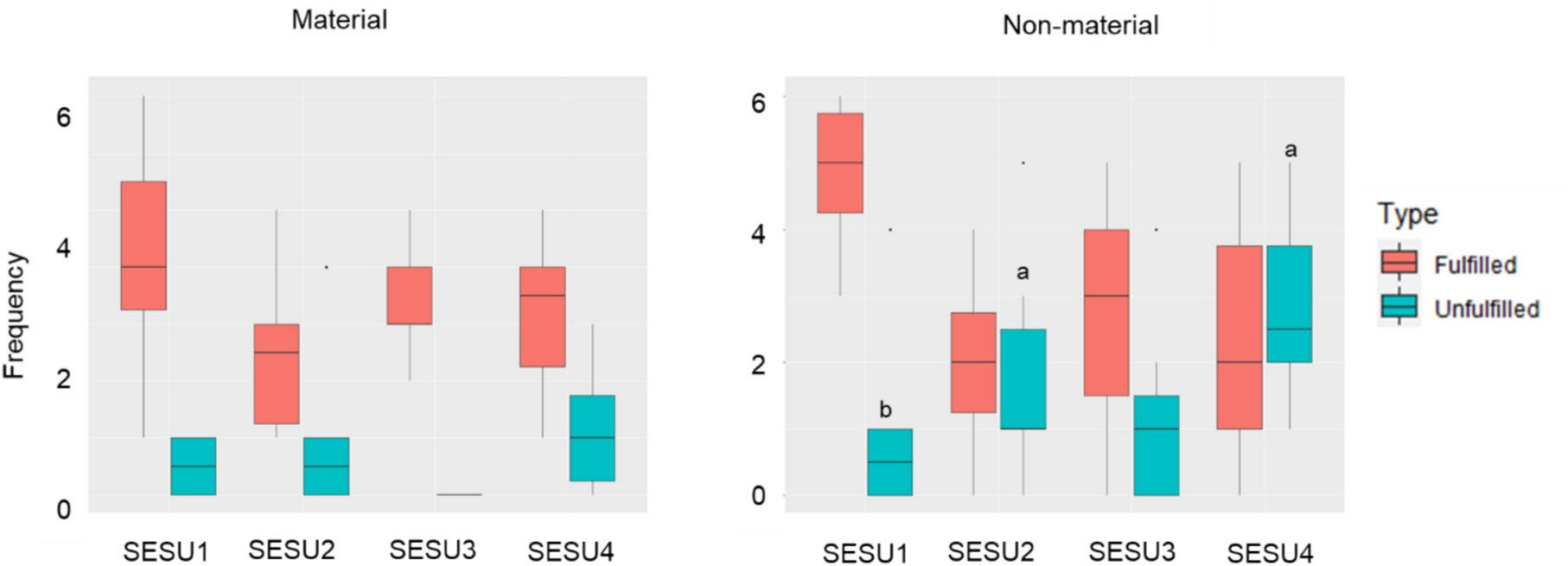
Changes in material and non-material fulfillment or unfulfillment among the four SESU. Lowercase letters indicate significant differences between SESU (Tukey posthoc test, p-value < 0.05)

The perception of fulfillment was linked with land transformation and governance. The perception of the fulfillment of non-material dimensions was highest in SESU1, with the lowest land transformation and lowest governance-driven individualism (Figs. 1 and 4). Conversely, the unfulfillment of non-material dimensions was highest for SESU4 and SESU2 compared to SESU1 (*F-statistic* = 3.69, df = 3, p-value = 0.027). We did not find significant differences in the fulfillment of material dimensions between SESU (*F-statistic* = 1.178, df = 3, p-value = 0.34, Table S6).

With these results we observed another vision, the “need to earn more”, specially illustrated in SESU4, where the governance practices rely on private land tenure, and smallholders expressed the highest ratio of unfulfilled material and non-material dimensions (Fig. 4). At the same time, smallholders of SESU4 were the ones who identified the highest number of obstacles to a good SWB (Fig. 3; Table 1). In SESU4, many smallholders whose *ejidos* are all located along the coast intend to increase their income by engaging in economic opportunities. For example, the quote, “*I would have liked to have more money to buy whatever I want, for instance, a car*” (S17, SESU4) illustrates the demand for more material goods. Smallholders in SESU4 mostly expressed instrumental values in arguments about the utility of nature for people, as for example with the quote “*I still have many things to do, I have the ambition to have more things, in my opinion, I am never fulfilled, I always want more*” (S20, SESU4).

## Discussion

This study assessed the SWB of smallholders who inhabit a biodiversity hotspot area by analyzing how subjective perceptions of wellbeing differed across SES contexts. In the following, we discuss (1) how multiple SWB dimensions maintain a sense of good wellbeing and how enablers and obstacles relate to that, (2) the role of land transformation, management intensity, and governance in fulfilling a sense of good life, (3) the strengths and limitations of this study, and (4) its implications for decision-making.

### A sense of a good subjective wellbeing: an intricate fabric of different dimensions and value systems

We found that SWB can be expressed in multiple ways (a total of six dimensions encompassing 48 different items) to describe the intimate relationship between the SWB of smallholders and the SES they inhabit and their perceptions of life. These dimensions are consistent with former typologies of SWB, (e.g., Rogers et al. 2012; Breslow et al. 2016), that highlight the value of material richness, work, and social relations, and emphasize the role of self-determination as one key dimension. Our results place at the top the importance of social capital (small community) highlighting the contribution of family bonding and friends in sustaining relationships that nurture a life worth living. Our results confirm the importance of relational values, the importance ascribed to nature because of its contribution to meaningful relations (Chan et al. 2016). Economic prosperity was second, making visible the role of nature through instrumental values, nature as a means to provide benefits to people (Arias-Arévalo et al. 2018; Díaz et al. 2015). The relevance of instrumental values that underpin the desire for a good SWB has also been recognized in the Northeast and Southern Thailand, where people regarded “money and assets” as the most important aspect of people’s lives (McGregor et al. 2009), and in the Caatinga dry forest of northeast Brazil (Dawson et al. 2021). Jobs were the third most important factor, echoing the recent adamant call by Brondizio et al. (2023) on the rapid decline of food producing jobs across the planet, causing the breakdown of families and communities. A combination of relational and instrumental values, underpinned by a sustained source of income from nature echo the desires for wellbeing across contrasting contexts in Switzerland as well as Bolivia (Chapman and Deplazes-Zemp 2023; Ortiz-Przychodzka et al. 2023).

One novel contribution of this paper is addressing the perceived obstacles and enablers to a good SWB. Beyond positive, maybe naive or romanticized perspectives of the advantages of peaceful enjoyable rural life (e.g. McGregor et al. 2009; Fagerholm et al. 2020), this paper emphasized the struggles faced by smallholders, mostly the older smallholders, that contribute to the sense of decadence and negativity towards life (Tauro 2019). Historically, peasants around the world, and in particular in the Global South, have historically faced marginalization, scarcity, and few opportunities for securing wellbeing (Davies 2008; Buciega et al. 2009).

### Living well vs. the need to earn more: how land transformation, management intensity, and governance shape subjective wellbeing

Connecting the way the landscape has changed, the way smallholders decide to manage their land, and the way wellbeing is perceived has seldom been addressed. Three interacting gradients across the territory—land cover transformation, low to high management intensity, and individual to collective governance (Santillán-Carvantes et al. 2023)—yielded different configurations of SWB. These different complex interactions can be summarized into two contrasting visions, “living well” or “need to earn more”.

Under the vision of “living well”, smallholders feel more content, perceived less obstacles to live a fulfilled life, and express more relational values since social capital (bonds with neighbors and family) was the most important component of wellbeing. This vision was linked to collective decision making, low management intensity and higher levels of biodiversity conservation. This “living well” perspective is associated with those isolated ejidos, with low access to markets for their products and the poorest households but also those with the strongest ties to Indigenous ancestors (Monroy-Sais et al. 2018, 2022; Balvanera et al. 2021), and a higher maintenance of forest cover (Santillán-Carvantes et al. 2023). The vision of “living well” is rooted in family and collective action (Bartra 2002), where a communal sense of community favors a sense of belonging, where people who know their neighbors are more likely to engage in activities that foster mutual support and collective leisure activities (Arana and Wittek 2016). This vision is in alignment with other perspectives of living in harmony with nature, such as the *Buen Vivir* in South America (Albó 2018), *Ubuntu* in sub-Saharan Africa (Chibvongodze 2016) and *Satoyama* in Japan (Torralba et al. 2023).

At the other extreme, under the vision of “need to earn more”, economic capital is the most privileged component of SWB, where land ownership is privatized and individual decisions prevail, management is more intensive and forest cover is rapidly disappearing. This vision is expressed by those smallholders who perceived more obstacles to fulfill their lives, who are mainly placed in those ejidos close to the highway. Those ejidos that are closer to the road have the best conditions for intensifying their management and have the easiest access to governmental support to foster cattle ranching (Mora et al. 2016; Balvanera et al. 2021). Moreover, the road offers them the opportunity of tourism development, leading to more job opportunities (including ecotouristic activities and employment in hotels), income increase, and access to education and health services; altogether achieved at the cost of nature and social cohesion (Riensche et al. 2019). These ejidos are now immersed into the dominant logic of cattle ranching as the accumulation of land and money (Torales-Ayala 2015; Hoelle 2018), leading to unsustainable production and consumption patterns and to a perpetual cycle of seeking material fulfillment through external means (Eckersley 2006).

The shift from “living well” to “earning more” is not only driven by the contrasting dynamics supported by the road and tourism, but also by national (and global) policies (Balvanera et al. 2021). The dominant narrative of the Program for Certification of Ejido Rights and Titling of Urban Plots (PROCEDE), the North American Free Trade Agreement (NAFTA) and its updated recent United States-Mexico-Canada Agreement (USMCA) is to provide commodities for global markets, overemphasizing material wellbeing at the cost of biodiversity conservation and non-material dimensions of wellbeing (Toledo 1996; Simon 2018).

### Strengths and limitations of our approach

In this paper, we applied a novel approach to explore SWB: we used in-depth interviews, and deductive-inductive content analysis to explore the role of land transformation, management intensity, and governance on wellbeing perceptions, for an understudied region in the Global South and for an understudied stakeholder: smallholders. We go beyond the common methodologies used to analyze SWB (OECD 2013; Millán and Castellanos 2018) and explore what people expressed beyond simple frequencies associated with numeric answers. In particular, qualitative methods such as content analysis have proved to be a useful approach to deeper understand social perceptions and preferences through the identification of values, concerns and motivations of local communities (Iniesta-Arandia et al. 2014; Quintas-Soriano et al. 2023). Our results revealed unexplored subjective dimensions of wellbeing such as the expressions of obstacles to reach wellbeing. The tradeoff in these in-depth explorations is that they have a limited statistical power, allowing only for exploratory rather than conclusive outcomes. Furthermore, despite this research did not consider in its goals the subjective nature of self-reported data and the potential influence of social desirability bias, further research will benefit from taking those aspects into account since a positive association has been shown between social desirability and expressed life satisfaction (Caputo 2017).

The *sense of decadence* that was unraveled by this study may have been biased by the gender and age of respondents: they all were smallholders; men and their average age was 60 years with some illness or a hard moment in their life. We chose to interview this profile of smallholders because they represent the current configuration of the ejidatario population across the region (Tauro et al. 2018, Tauro 2019). The lack of younger ejidatarios and women is explained by the increasing rates of outmigration of youth across many rural areas of the country (Jiménez-Moreno et al. 2023), and the law configuration largely excluding women from right to land (Herrera Rodriguez 2012). Nevertheless, in this study, we intended to emphasize differences across ejidos (and SESU) instead of within an ejido. Therefore, older men are not only the most representative population of the ejido but also the representatives of decision-making in the ejidal assembly. Further explorations of intersectional perspectives would be needed in order to consider women and other age sectors.

The expression of feelings of sadness, anger, stress, or pain have been identified as of a “hedonistic” wellbeing alongside other emotions such as happiness (Steptoe et al. 2015). Wellbeing from these psychological aspects contains both positive and negative dimensions that provide unique information about a person’s emotional state (Steptoe et al. 2015). Thus, wellbeing expressed through emotions, along with life satisfaction (cognitive aspects) and purposes, allows for evaluating the multidimensionality of SWB in specific conditions and contexts. Men in rural areas are more vulnerable to experiencing extreme sadness due to the hegemonic masculinity in rurality, which has served men well in past times, allowing them power and privilege, but unhealthy in times of significant stress (Narayan et al. 2002). The constant droughts in the Chamela-Cuixmala region, the inequities in access to markets and labor, the lack of access to public health services, the absence of rural insurance against climate disasters, and limited economic support for productive activities increase pressures on smallholders, leading to unpleasant emotions and lower satisfaction levels with life.

### Implications for decision-making for tropical dry forests

The way nature is valued in political and economic decisions is both a key driver of the global biodiversity crisis and a vital opportunity to address it (IPBES 2022). To develop effective intervention programs that benefit the wellbeing of diverse communities, it is essential to deeply address the way people perceive and value nature and how its wellbeing depends on it, and explicitly include their diverse perspectives in decision-making (Bartra 2002; Tauro et al. 2018; IPBES 2022). The two SESU with wellbeing models based on opposing views—“living well” vs. “the need to earn more”—exemplifies how the relation of people with nature differ under different social-ecological contexts shaped by governance systems. Our findings suggest that public policies in western Mexico could better support the wellbeing of smallholders, and the sustainable management of the large diversity found there, by nurturing social interactions and cohesion, rather than economic growth and individual material gains. For instance, programs such as PROCAMPO (support to agriculture), and PROGAN (support to cattle ranching) could contribute to securing public health programs and strengthening collective organization, rather than focusing on per hectare productivity.

## Conclusion

This study provides a comprehensive assessment of the subjective wellbeing of smallholders across different socio-ecological systems located in a biodiversity hotspot in the western pacific coast of Mexico. Results highlighted the importance of social capital, economic prosperity, and meaningful employment for subjective wellbeing. Our study also evidenced the two prevailing visions of subjective wellbeing in different tropical dry forests in western Mexico; the vision of ‘living well’ prevails, especially in areas with communal governance and medium management intensity, while the vision of “need to earn more” prevails in areas of individual governance and intensified land management, associated with the land market for coastal tourism development. Our findings illustrate the complex interplay that relational and instrumental values play in shaping perceptions of smallholders’ wellbeing. We call the need for policies that promote social cohesion and communal values over purely economic growth. With these results, we advanced the understanding of subjective wellbeing dimensions by people in rural areas of the Global South and highlighted the diversity of worldviews of what is considered a life worth living. Future research in the subject could include understanding deeper the effect of policies on subjective wellbeing, addressing potential social desirability bias, incorporating quantitative methods alongside qualitative approaches and inclusion of a more diverse sample, encompassing different genders and age groups, to capture a wider range of perspectives on subjective wellbeing.

## Supplementary Information

Below is the link to the electronic supplementary material.

Supplementary file1 (DOCX 42 KB)

## Data Availability

The dataset used during the current study are publicly available at 10.13140/RG.2.2.15550.70724.
