# Supplementary material for: Impact of land transformation, management and governance on subjective wellbeing across social–ecological systems
Source: Sustain Sci. 2024 Dec 12;20(2):469–83. doi: 10.1007/s11625-024-01584-5 (PMC11937195; doi:10.1007/s11625-024-01584-5)
Supplement: Supplementary file 1 — Supplementary file1 (DOCX 42 KB) [file 11625_2024_1584_MOESM1_ESM.docx]

Electronic Supplementary Material

Table S1. Subjective wellbeing (SWB) dimensions, obstacles and enablers, and their frequencies in the Chamela-Cuixmala Region

| **Dimension** | **Subdimension** | **Frequency** | **Example** |
| --- | --- | --- | --- |
| **Social capital** | Small community | 39 | “I love my children” “I am happy” “I have my family” |
|  | Social interaction | 22 | “My daughter supports me with the visits to the doctor” “respect form others”, “speak to get along with people”, “[parties occur] at the expense of a group of people that cooperates”, “don’t get into what you don’t care about” |
|  | Future Generations | 15 | “Children admire their parents” |
|  | Cohesion | 16 | “They [his children] can collaborate with each other”, “his children call him to be pendant about him”, “I have a peaceable family”, “leave everything, at work in order to help my family”, “helping people who needs information” |
|  | Traditions | 9 | “Respect from others”, “I have a peaceable family”, “children admire their parents”, “according to what you do, people will respect you”, “when there’s money, there’ll be a party”, “[you perform] the gift that God give you”, “people respect me”, “if your son likes livestock, he should work on it” |
|  | Cultural heritage | 4 | “I appreciate beautiful things” |
|  | Nurturing childhood | 2 | “I bought a house in Colima, where me and my sons live and afterwards his other daughters will arrive”, “giving advice to your children since they are little” |
| **Economic capital** | Job | 43 | “it’s fine, I have a job”,  “it’s fine, I work in an independent way” “it’s fine, I have a job, an income” |
|  | Economic prosperity (having economic capital) | 41 | “I want to make progress”, I bought a house”, “we [me and my family] have savings”, “these days I have been working and having good results” |
|  | Work place | 14 | “When I go to the paddock I feel good, I work a little, I don’t make a big effort, but going to the paddock works as a distraction, and I am not staying all the day at home”, “I can work in my patch”, “I don’t want to sell my land and animals because it gives me illusion to having them”, “building a house in order to start a business (in the town) and not coming to the paddock and working hard” |
|  | Job security | 6 | “I am tired, but fine because I have holidays and I can work on my land, I want to make the most out of my time”, it’s fine, I have a job, an income”, “we have to work hard, make it better, to live in a better way, having a good nutrition, an income, a job, if there’s no job, there’s no money, we need to increase livestock, the number of cows and pasture” |
| **Agency** | Freedom | 28 | “I am always doing something”, “I work independently, there’s no schedule”, “I take care of my own land” |
|  | Ambition to have more | 12 | “Keeping the cows, but having more cattle", "I still have things to do, I aspire to do more things in order to get more material goods, from my view I never get enough, I always wants more projects [in relation to goods]", "I would like more of what I have, but I can’t, you can’t change your future” |
|  | Motivation and intention to live | 11 | “I like this life”, “I want to take advantage of time to the maximum”, “with plans for a one-thousand years future” |
|  | Mobility | 12 | “I can move freely”, “I walk all day” |
|  | Sense of responsibilities | 6 | “Consuming what we produce”, “having responsibilities”, “free yourself from the duty to provide information about the *ejido*...disengage |
| **Nature** | Organisms (positive perspective) | 15 | “I feel happy and my animals have good food. That motivates me” |
|  | Scenery | 14 | “Before I went to the plot by horse”, “with extra money he can hang out with his children (to the cinema, beach, football)”, “I see the landscapes” |
|  | Observe nature | 10 | “I have green land”, “the vegetation”, “to sow”, “I like the hill [word for the Spanish *Monte,* in reference to the tropical dry forest”] |
|  | Water | 5 | “[I would like that] rain doesn’t lack”, “[I would like that] it wouldn’t stop raining, to get future”, “Me and my family are healthy, as long as there is rain, water, work, if it misses the rain, there is no work” |
|  | Geographic features | 3 | “I am happy in the field, seeing the animals, the hill, I like the hill” [word for the Spanish *Monte,* in reference to the tropical dry forest”] |
|  | Forests | 2 | “don’t cut down forests, keep some useless parts as paddock, it helps to vegetation, preserve *guayabillo* wood”, “to plant more trees and maintain them, to use forest in a sustainable way” |
|  | Pleasant weather | 2 | “We cheer us up; we made a mistake coming here. Where we come from the weather is fine, occurs agriculture, we came of thinking the land was able for agriculture, at the beginning was tough, we became ranchers, I wanted to be farmer, [in our previous town] we had 8 ha, here they offered to us 35 ha per *ejidatario”* |
|  | Closeness to nature | 1 | “I like livestock a lot, I live from it… all the field seems such a beautiful thing to me” |
| **Pleasant non-work activities** | Other outdoor activities | 9 | “going to the paddock serve me as a distraction… not being at home all day..., it makes me sleepy to be at home” |
|  | Leisure | 8 | “I have work to do, with extra money I can hang out with his children (to the cinema, beach, football), to spend time with my family”, “I couldn’t answer well, life gives you some pleasant times and others… life offers everything”, “not having the need for working, living in a comfortable place, having fun” |
|  | Tourism | 3 | “Start a ecotourism project in the *ejido* fields, interpretative trails, adventure tourism... we want to be the first in the region” |
| **Government and services** | Private services | 6 | “[having] a good mode of transport”, having a good car” |
|  | Accountable government | 5 | “in the public scenario, a change in the government, there is no future for the people below…”, “that the government doesn’t steal money from people”, “in politics, to have a nice government” |
|  | Public services (electricity, water, education) | 5 | “Medical appointments”, “I am being attended for a doctor from Guadalajara”, “before I went to the plot by horse, now I go by car” |
|  | Public security | 1 | “In more than 10 years I am thinking about taking refuge with the daughters even if they live in a big town”, “health and security” |
| **Obstacles** | Sense of decadence | 24 | “I would like God give me life without getting old”, “I am wasted, I can’t work, I get tired quickly”, “cruel because I lost my youth”, “I didn’t know how to take advantage of past opportunities”, “I was wealthy and I spent it away”, it is difficult to describe [a good life], I live in lack”, “not come to the paddock to work sacrificially”, “for people in their 50 or 60 years old there is no way to live fully anymore, there is no space for wishes”, “at this age you can’t aspire to have something, a person can’t work, or keep what you have” |
|  | Awareness of self-limitations | 18 | “To be aware about what you do wrong”, “life depends on external forces”, “we have to deal with life as it comes” |
|  | Bad quality of life | 11 | “insecurity generates me worries”, everything is suffering”, “withered, without hopes”, “I doesn’t feel safe [in life]”, “wasted, I worked hard”, “it’s only working”, “I would have liked that God gave me life without getting old”, “I can’t thing or imagine the good life”, “due to my age I can’t work anymore, but I don’t want to receive alms” |
|  | Bad health | 9 | “Exhausted, tired”, “old man, everything is suffering”, “I would have liked that God gave me life without getting old” |
|  | Sense of scarcity | 7 | “I was wealthy and I spent it away”, “what does oneself? pure work”, “sacrifice” |
|  | Sense of concern | 4 | “life is a function of external forces, I feel disappointed, insecurity generates me concern. something is not working well, but you don’t have to make your life miserable for that”, “I depend on family, at this age there is no job, if your family supports you… if they make you part of your own family, if they don’t, the alternative is to ask for charity” |
|  | Public insecurity | 4 | “Here, I enjoy my life, but I am dissatisfied because of the insecurity”, “what can I ask for? To eat and live peaceful, if they allow us. Our current situation [insecurity] is the problem, bosses can’t control that”, “we don’t know what our destiny is [in terms of insecurity]” |
|  | Organisms (negative perspective) | 2 | Sometimes I deny to take care of my animals, but what do I do? Pure work”, “to build a little house in order to start a business (in the village) and not going to the paddock and work hard” |
|  | Sadness | 2 | “What would I do...? to look up for a good life doesn’t fit with me, it’s tough to describe it, I have a life of misery”, “I don’t feel happy” |
| **Enablers** | Good health | 19 | “To be less tired”, “I am very good, fulfilled, I have a complete family, I am happy with my family, I work independently, without an schedule, I am healthy and strong”, “thank God, the world owes me nothing, I asked to God that my family doesn’t get sick and he gave it to me [ in the sense of drug addict children]” |
|  | Good food (for human and animals) | 18 | “I don’t buy grass”, “we enjoy what we eat” |
|  | Tranquillity | 18 | “To feel fine”, “little by little you make progress”, the world owes me nothing”, “I am fulfilled” |
|  | Happiness | 16 | “I am not upset for being in the world”, “I feel fine”, “I love my children” |
|  | Good quality of life | 10 | “The benefits level have raised”, “I am fulfilled”, “If I could, I would move forward”, “I am satisfied”, “being healthy allows me to work and have facilities”, “to be clean” |
|  | Sense of plenitude | 10 | “Really fine, completed” “fulfilled”, “yes [I am very satisfied with my life] because I have reached this period in my life” |
|  | Housing | 6 | “To have the necessary: such as a car, which I can go to the city with. A nice house, with services, well painted and finished”, “that nothing is missing in the house, not having illness", "getting off from work, have a house on the hill (on your plot), to dedicate yourself to cattle" |
|  | Sense of place | 5 | “I enjoy staying in the village”, “he feels fine with all people” |

| Table S2. Data set with the information of the smallholders | | | | | | | | |
| --- | --- | --- | --- | --- | --- | --- | --- | --- |
| **Smallholder** | **SESU** | **Education** | **Material**  **Fulfilled** | **Material**  **Unfulfilled** | **non-material**  **Fulfilled** | **non-material**  **Unfulfilled** | **Age** | **Years living in the zone** |
| S1 | SESU3 | primaria | 3 | 1 | 4 | 4 | 72 | 30 |
| S2 | SESU3 | primaria | 1 | 1 | 4 | 1 | 34 | 34 |
| S3 | SESU4 | sin escuela | 3 | 0 | 4 | 1 | 77 | 40 |
| S4 | SESU2 | sin escuela | 5 | 3 | 1 | 5 | 79 | 60 |
| S5 | SESU3 | primaria | 1 | 0 | 6 | 0 | 63 | 38 |
| S6 | SESU2 | primaria | 7 | 0 | 5 | 4 | 50 | 41 |
| S7 | SESU2 | primaria | 4 | 0 | 0 | 2 | 54 | 50 |
| S8 | SESU3 | secundaria | 1 | 0 | 1 | 1 | 55 | 38 |
| S9 | SESU2 | primaria | 5 | 0 | 2 | 3 | 40 | 40 |
| S10 | SESU1 | secundaria | 4 | 0 | 5 | 0 | 54 | 46 |
| S11 | SESU1 | primaria | 2 | 0 | 2 | 0 | 53 | 48 |
| S12 | SESU4 | sin escuela | 3 | 1 | 3 | 0 | 77 | 60 |
| S13 | SESU2 | primaria | 3 | 0 | 0 | 4 | 74 | 25 |
| S14 | SESU1 | primaria | 5 | 0 | 3 | 2 | 67 | 66 |
| S15 | SESU4 | preparatoria | 4 | 1 | 4 | 1 | 40 | 40 |
| S16 | SESU1 | preparatoria | 3 | 0 | 1 | 0 | 69 | 48 |
| S17 | SESU4 | sin escuela | 2 | 0 | 2 | 0 | 73 | 44 |
| S18 | SESU2 | secundaria | 3 | 0 | 3 | 1 | 50 | 45 |
| S19 | SESU3 | sin escuela | 4 | 0 | 4 | 1 | 59 | 35 |
| S20 | SESU4 | primaria | 1 | 4 | 1 | 5 | 76 | 15 |
| S21 | SESU1 | primaria | 3 | 1 | 0 | 1 | 59 | 45 |
| S22 | SESU4 | primaria | 6 | 0 | 6 | 1 | 44 | 44 |
| S23 | SESU1 | secundaria | 2 | 2 | 3 | 2 | 62 | 33 |
| S24 | SESU3 | sin escuela | 4 | 1 | 5 | 0 | 70 | 70 |
| S25 | SESU3 | primaria | 4 | 1 | 5 | 3 | 60 | 53 |

| **Table S2.** Continuation Data set with the information of the smallholders | | | | | | | | | | |
| --- | --- | --- | --- | --- | --- | --- | --- | --- | --- | --- |
| **Smallholder** | **SESU** | **Diversity of income sources** | **Number of land rights** | **Number of owned plots** | **Number of cattle** | **Number of federal programs support that they receive** | **Assistance to the doctor in the last year** | **Facility of access to water** | **Reception of remittances?** |  |
| S1 | SESU3 | 1 | 2 | 1 | 100 | 1 | 1 | Hard | yes |  |
| S2 | SESU3 | 2 | 1 | 1 | 15 | 0 | 1 | Medium | no |  |
| S3 | SESU4 | 1 | 1 | 4 | 30 | 2 | 1 | Hard | no |  |
| S4 | SESU2 | 1 | 1 | 1 | 24 | 0 | 0 | Easy | yes |  |
| S5 | SESU3 | 1 | 1 | 2 | 4 | 1 | 2 | Medium | no |  |
| S6 | SESU2 | 3 | 1 | 1 | 40 | 1 | 1 | Easy | no |  |
| S7 | SESU2 | 3 | 1 | 7 | 100 | 1 | 2 | Easy | no |  |
| S8 | SESU3 | 2 | 1 | 1 | 20 | 0 | 0 | Medium | yes |  |
| S9 | SESU2 | 2 | 1 | 5 | 50 | 0 | 0 | Medium | no |  |
| S10 | SESU1 | 1 | 1 | 3 | 19 | 1 | 2 | Easy | no |  |
| S11 | SESU1 | 4 | 2 | 4 | 60 | 1 | 1 | Easy | no |  |
| S12 | SESU4 | 1 | 1 | 1 | 40 | 1 | 1 | Easy | no |  |
| S13 | SESU2 | 1 | 1 | 2 | 230 | 2 | 0 | Hard | no |  |
| S14 | SESU1 | 2 | 1 | 2 | 35 | 1 | 3 | Medium | no |  |
| S15 | SESU4 | 2 | 1 | 2 | 57 | 2 | 1 | Easy | no |  |
| S16 | SESU1 | 2 | 1 | 4 | 58 | 0 | 3 | Easy | no |  |
| S17 | SESU4 | 1 | 1 | 1 | 120 | 1 | 3 | Easy | yes |  |
| S18 | SESU2 | 2 | 1 | 8 | 120 | 1 | 2 | Easy | no |  |
| S19 | SESU3 | 1 | 1 | 3 | 40 | 1 | 1 | Hard | no |  |
| S20 | SESU4 | 1 | 1 | 1 | 70 | 1 | 1 | Medium | yes |  |
| S21 | SESU1 | 2 | 1 | 3 | 35 | 1 | 2 | Medium | yes |  |
| S22 | SESU4 | 3 | 1 | 1 | 20 | 1 | 2 | Easy | no |  |
| S23 | SESU1 | 2 | 1 | 11 | 300 | 1 | 3 | Medium | no |  |
| S24 | SESU3 | 1 | 1 | 2 | 30 | 2 | 0 | Medium | no |  |
| S25 | SESU3 | 3 | 1 | 4 | 50 | 1 | 1 | Hard | no |  |

**Table S3.** Differences per current SWB dimensions and obstacles and enablers per SESU

|  | **p** | **F-value/X^2^** | **Df** |
| --- | --- | --- | --- |
| Economic capital | 0.147 | F= 1.98 | 3 |
| Social capital | 0.978 | X^2^=0.195 | 3 |
| Agency | 0.938 | X^2^=0.41 | 3 |
| Nature | 0.82 | X^2^=0.92 | 3 |
| Pleasant non-work activities | 0.125 | X^2^=5.72 | 3 |
| Government and services | 0.326 | X^2^=3.457 | 3 |
| Obstacles | 0.209 | X^2^=4.53 | 3 |
| Enablers | 0.687 | X^2^=1.47 | 3 |

**Table S4.** Differences per desirable SWB dimensions and obstacles and enablers per SESU

|  | **p** | **F-value/X^2^** | **Df** |
| --- | --- | --- | --- |
| Economic capital | 0.57 | X^2^=2.002 | 3 |
| Social capital | 0.524 | X^2^=2.239 | 3 |
| Agency | 0.713 | X^2^=1.367 | 3 |
| Nature | 0.296 | X^2^=3.692 | 3 |
| Pleasant non-work activities | 0.27 | X^2^=3.885 | 3 |
| Government and services | 0.709 | X^2^=1.385 | 3 |
| Obstacles | 0.413 | X^2^=2.86 | 3 |
| Enablers | 0.1 | F= 2.365 | 3 |

**Table S5**. T-test/ Mann Whitney between current and desire SWB

| **SESU1** | **p** | **W/T** | **Df** |
| --- | --- | --- | --- |
| Economic capital | 0.241 | T= 1.245 | 10 |
| Social capital | 0.08 | 29 |  |
| Agency | 0.562 | 22 |  |
| Nature | 0.731 | 20.5 |  |
| Pleasant non-work activities | 0.44 | 23 |  |
| Government and services | 0.246 | 25 |  |
| Obstacles | 0.6 | 21 |  |
| Enablers | 0.002 | T=3.97 | 10 |
| **SESU2** | **p** | **W** | **Df** |
| Economic capital | 0.45 | T=0.785 | 10 |
| Social capital | 0.366 | 24 |  |
| Agency | 0.067 | 29.5 |  |
| Nature | 0.234 | T=1.264 | 10 |
| Pleasant non-work activities | 1 | 18 |  |
| Government and services | 1 | 18 |  |
| Obstacles | 0.446 | 23 |  |
| Enablers | 0.325 | 24.5 |  |
| **SESU3** | **p** | **W** | **Df** |
| Economic capital | 0.189 | T=1.391 | 12 |
| Social capital | 0.077 | 38.5 |  |
| Agency | 0.07 | 38 |  |
| Nature | 0.18 | 34 |  |
| Pleasant non-work activities | 1 | 24.5 |  |
| Government and services | 0.783 | 27 |  |
| Obstacles | 0.411 | 31 |  |
| Enablers | 0.002* | T=3.739 | 12 |
| **SESU4** | **p** | **W** | **Df** |
| Economic capital | 0.139 | T=1.604 | 10 |
| Social capital | 0.623 | 21.5 |  |
| Agency | 0.587 | T=0.561 | 10 |
| Nature | 0.822 | T=0.23 | 10 |
| Pleasant non-work activities | 0.929 | 19 |  |
| Government and services | 0.787 | 20 |  |
| Obstacles | 0.181 | T=1.435 | 10 |
| Enablers | 0.885 | T=0.147 | 10 |

**Table S6.** Differences between material fulfilled-unfulfilled per SESU

| **Material** | **p** | **F-value/X^2^** | **Df** |
| --- | --- | --- | --- |
| Fulfilled | 0.342 | F= 1.178 | 3 |
| Unfulfilled | 0.081 | X^2^=6.72 | 3 |
| **Non-material** |  |  |  |
| Fulfilled | 0.027* | F=3.69 | 3 |
| Unfulfilled | 0.11 | X^2^= 5.97 | 3 |

Tukey HSD Non-material, fulfilled

SESU1-SESU2 p= 0.03; SESU1-SESU4 p= 0.05
